# A Three-Year-Old Child With Recurrent Wheezy Chest: An Unusual Presentation of Thoracic Neuroblastoma

**DOI:** 10.7759/cureus.108594

**Published:** 2026-05-10

**Authors:** Julia Qamar Bilalaga, Amr Khawatmi, Nagam AlShehabi, Mouna Hathat, Rafia Owera

**Affiliations:** 1 Medicine, University Hospital Sharjah, Sharjah, ARE; 2 Medicine, George Emil Palade Universitatea de Medicină, Farmacie, Științe și Tehnologie, Targu Mures, ROU; 3 Medicine, Mohammed Bin Rashid University of Medicine and Health Sciences, Dubai, ARE; 4 Medicine, Gulf Medical University, Ajman, ARE; 5 Pediatrics, Prime Medical Center, Dubai, ARE

**Keywords:** mediastinal mass, pediatric oncology, pediatric respiratory symptoms, recurrent wheeze, thoracic neuroblastoma

## Abstract

We present the case of a three-year-old child with a history of recurrent wheezy chest and lower respiratory tract infections, who was initially managed as having recurrent viral-induced wheeze. However, persistent symptoms and poor response to standard treatment prompted further investigations, which eventually led to a diagnosis of thoracic neuroblastoma. This case highlights the importance of considering alternative diagnoses in children with recurrent respiratory symptoms, especially when not responding to conventional therapy.

## Introduction

Neuroblastoma is an embryonal tumor of the sympathetic nervous system and represents the most common extracranial solid malignancy in childhood. It can arise anywhere along the sympathetic chain, most frequently in the adrenal glands. However, other sites, including the thorax, neck, pelvis, and rarely organs such as the kidney or thymus, have been reported. Clinical presentation varies widely depending on tumor location and stage, ranging from abdominal distension and neurological deficits to incidental radiologic findings [1].

Risk stratification is based on histopathological features, molecular markers, and disease stage, which together guide therapeutic decisions. Management ranges from surgical resection alone in low-risk disease to multimodal therapy, including chemotherapy, immunotherapy, and hematopoietic stem cell transplantation, in higher-risk groups [1]. Notably, neuroblastic tumors exist along a biological spectrum, with some lesions demonstrating spontaneous regression or maturation into ganglioneuroma [2].

Thoracic or mediastinal neuroblastomas represent a minority of all neuroblastoma cases. They may present incidentally or with respiratory or neurologic manifestations, particularly when associated with airway compression or spinal involvement [3], and generally carry a more favorable prognosis [4]. These respiratory symptoms can mimic common pediatric conditions such as asthma or recurrent lower respiratory tract infections.

Although respiratory symptoms have been described in thoracic neuroblastoma, wheezing as a predominant presenting feature is uncommon. This case highlights the importance of maintaining a broad differential diagnosis in children with persistent or atypical respiratory symptoms, particularly when radiologic findings do not resolve as expected, and supports consideration of underlying intra-thoracic masses as part of that evaluation.

## Case presentation

A three-year-old girl was seen in the pediatric clinic in Dubai, having recently arrived with her parents from Egypt. At this initial visit, the parents reported that her primary symptoms included a productive cough with thick secretions, chest congestion, intermittent wheezing, tachypnea on exertion, and a low-grade fever. She had been previously diagnosed as having mild persistent asthma, characterized by recurrent episodes of dry cough, wheezy breathing, and occasional difficulty in respiration, and had been prescribed nightly Montelukast 4 mg chewable tablets as prophylaxis. There was no family history of respiratory or skin allergies, no history of consanguinity, and she was the only child. The child had not required prior hospital admissions and was otherwise healthy, with no history of malnutrition, anemia, or musculoskeletal problems.

Upon examination, the child weighed 22.5 kg, had a height of 109 cm, and a BMI of 18.4. Vital signs were stable with a temperature of 37°C, respiratory rate of 20 breaths per minute, heart rate of 70 beats per minute, and oxygen saturation of 98% on room air. Chest auscultation revealed bilateral wheezing and crepitations, with reduced air entry over the right lower lung field. No cyanosis, pallor, lymphadenopathy, or organomegaly was noted. Other systemic examinations, including cardiovascular, abdominal, musculoskeletal, skin, and neurologic, were unremarkable.

Laboratory investigations revealed a complete blood count (CBC) with hemoglobin, red blood cells, and platelets within normal limits. Neutrophil and eosinophil counts were mildly elevated, suggestive of airway inflammation or an allergic process. C-reactive protein (CRP) was 1 mg/L (reference range: 0-5 mg/L), consistent with the absence of systemic bacterial infection. Influenza A and B tests were negative. These results are summarized in Table 1.

**Table 1:** Laboratory results CRP: C-reactive protein; MCHC: mean corpuscular hemoglobin concentration; RDW: red blood cell distribution width; MCV: mean corpuscular volume; MCH: mean corpuscular hemoglobin

Test	Patient Value	Reference Range	Interpretation
RBC Count	4.89 ×10¹²/L	4.0 – 5.2 ×10¹²/L	Normal
Hemoglobin	12.9 g/dL	10.7 – 14.7 g/dL	Normal
Hematocrit	38%	34 – 40%	Normal
MCV	78 fL	75 – 87 fL	Normal (slightly low-normal)
MCH	26 pg	24 – 30 pg	Normal
MCHC	34 g/dL	31 – 37 g/dL	Normal
RDW	13%	11.6 – 15.0%	Normal
Platelet Count	306 ×10⁹/L	170 – 450 ×10⁹/L	Normal
WBC Count	8.02 ×10⁹/L	5 – 15 ×10⁹/L	Normal
Neutrophils	55%	25 – 57%	Normal
Lymphocytes	32.70%	35 – 65%	Slightly low (may reflect mild stress or infection recovery)
Monocytes	8.95%	2 – 10%	Normal (upper range)
Eosinophils	2.42%	1 – 8%	Normal
Basophils	0.93%	0 – 2%	Normal
CRP	1 mg/L	0 – 5 mg/L	Normal (no active inflammation or infection)
Influenza A	Negative	—	No infection detected
Influenza B	Negative	—	No infection detected

Chest radiography demonstrated a right lower paracardiac opacity initially interpreted as consolidation, with a medial border inseparable from the cardiac silhouette. The working diagnosis included upper respiratory tract infection, acute bronchitis, and exacerbation of viral-induced wheeze (cough-variant asthma).

Treatment included intramuscular ceftriaxone (1000 mg once daily), oral clarithromycin syrup twice daily (7.5 mg/kg after food for 10 days) to cover atypical bacterial infection, a short course of corticosteroids, nebulized bronchodilators (salbutamol, budesonide, and normal saline), chest physiotherapy twice daily, and continuation of Montelukast for asthma control. Ten days later, her condition improved considerably, with resolution of cough and tachypnea, and her chest was clear on auscultation.

Follow-up chest radiography revealed persistence of the previously described opacity in the right lower paracardiac region (Figure 1). The parents were advised to obtain a chest CT scan and a pulmonology consultation, but they preferred to return to Egypt for ongoing care. Before traveling, the child received a Vaxigrip tetra influenza vaccine, as well as vitamin D and multivitamin supplementation.

**Figure 1:**
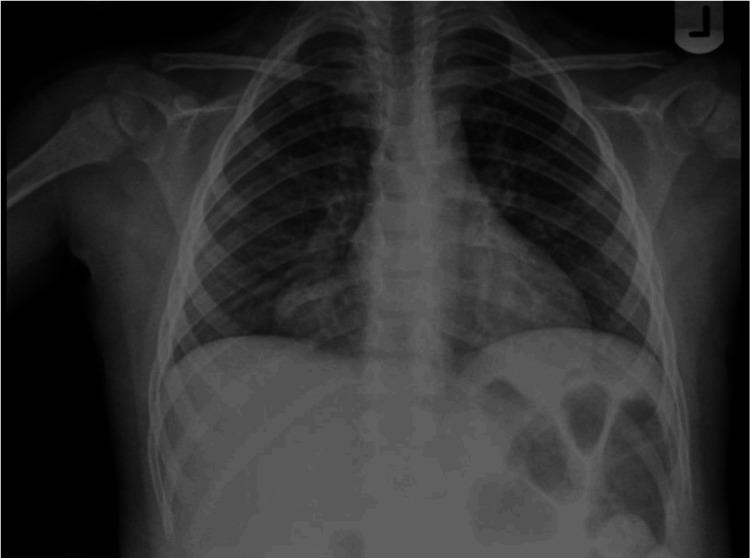
Chest X-ray (anteroposterior view) reveals a persistent round homogenous opacity in the right lower paracardiac region, inseparable from the cardiac silhouette.

Nearly a year later, she returned with similar complaints of recurrent cough, wheeze, congestion, and repeated "chest infections." The parents remained concerned about asthma. Follow-up chest radiography demonstrated persistence of the right paracardiac lesion with an interval increase in size. The lesion appeared as a well-defined round opacity along the right cardiac border, inseparable from the cardiac silhouette (Figure 2).

**Figure 2:**
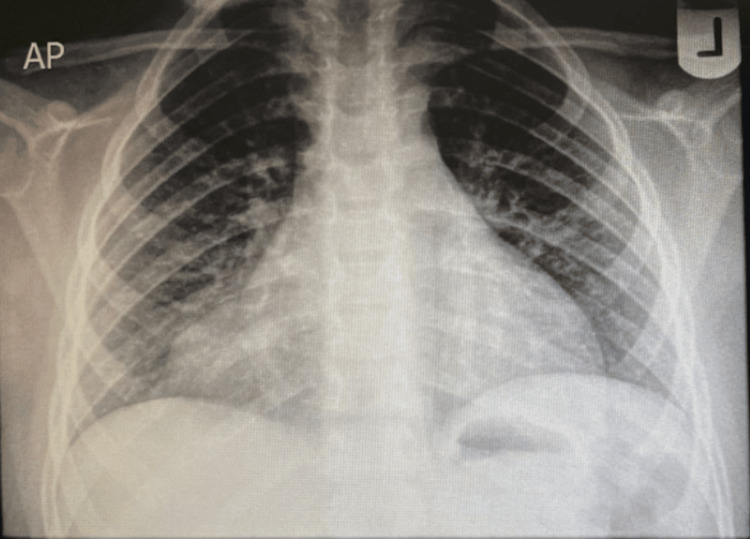
Repeat chest X-ray (anteroposterior view) nearly a year after initial presentation. Follow-up chest radiograph demonstrating a persistent right paracardiac opacity with an interval increase in size compared to prior imaging. The lesion appears as a well-defined, homogeneous round opacity along the right cardiac border, with loss of the adjacent cardiac silhouette.

On examination, she appeared unwell, with wheeze, crackles, decreased air entry on the right side, nasal and throat congestion, and mild tachypnea. She was once more treated symptomatically with bronchodilators, inhaled corticosteroids, nebulization, and physiotherapy. Despite some improvement, the opacity on the chest X-ray persisted. Again, a chest CT scan and pulmonology review were strongly advised, but the family opted to return to Egypt without undergoing further investigations.

The child returned once again with the same history, approximately four to five months after the second presentation, this time appearing more ill, with significant respiratory distress, persistent wheeze, and tachypnea. Repeat chest radiography demonstrated an increase in the homogeneous opacity in the right lower paracardiac region. Given the chronicity, lack of resolution, and imaging findings suggestive of airway compression as a contributor to the recurrent wheezing, a chest CT was insisted upon and performed locally in Dubai.

A high-resolution multi-slice CT scan revealed a well-defined ovoid mediastinal mass in the right paracardiac region, extending from the anterior to the middle mediastinum. The lesion showed heterogeneous enhancement with internal non-enhancing hypodense areas, consistent with cystic or necrotic components. The mass was noted to compress the right main bronchus and adjacent lung parenchyma, raising suspicion for a neoplastic process (Figure 3).

**Figure 3:**
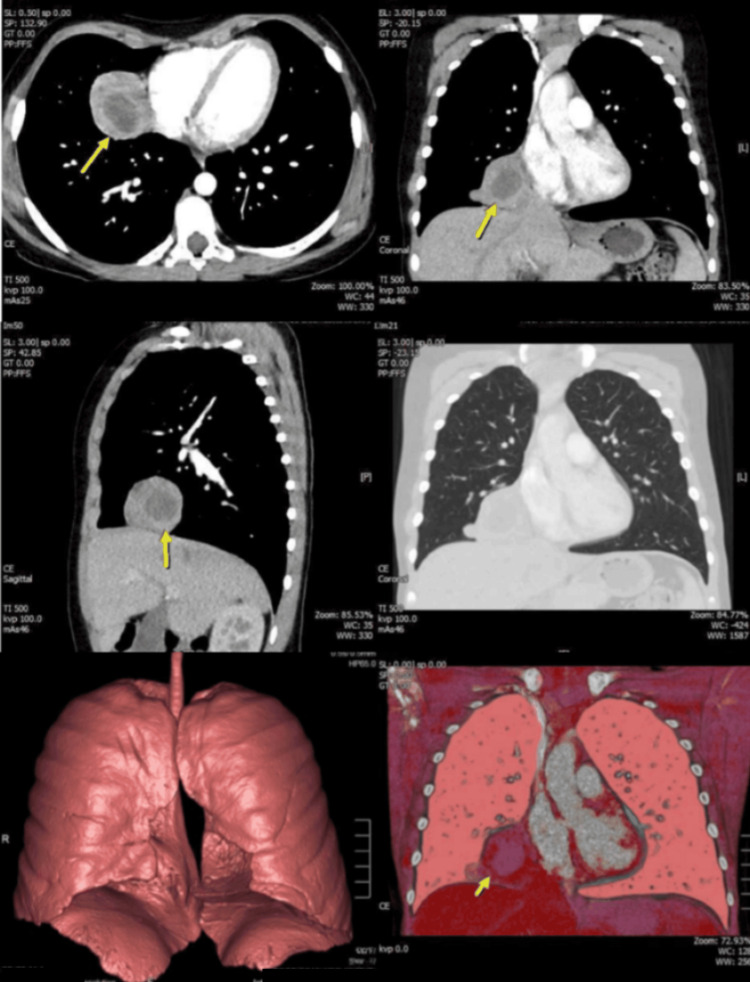
A high-resolution multi-slice CT of the chest at the third presentation CT of the chest reveals a well-defined ovoid soft tissue density in the right paracardiac region showing heterogeneous post-contrast enhancement with an internal non-enhancing hypodense cystic/necrotic component, measuring about 4 x 4.5 x 4 cm in maximum orthogonal dimensions; internal foci of faint calcifications are noted.

These findings prompted urgent referral to the pulmonology team, who recommended thoracic surgical evaluation, as bronchoscopy was deemed inappropriate in the context of a mediastinal mass. The patient underwent successful surgical excision of the mediastinal mass in Egypt. The postoperative course was uneventful, with complete resolution of cough, wheezing, and chest congestion.

Microscopic examination of the resected mass revealed sheets of small to medium-sized neuroblastic cells with round nuclei, fine chromatin, and indistinct cytoplasmic borders, arranged within a fibrillary (neuropil-rich) background. Foci of ganglion cell differentiation were identified. The mitotic-karyorrhectic index was low. These features are consistent with a partially differentiated neuroblastoma with favorable histology according to the Shimada classification [5]. Molecular testing for *MYCN* amplification using fluorescence in situ hybridization (FISH) revealed the absence of amplification.

Bone marrow examination demonstrated a normocellular marrow for age with normal trilineage hematopoiesis and no evidence of tumor infiltration. In conjunction with favorable histopathology and molecular testing, these findings supported a favorable prognostic profile. Metaiodobenzylguanidine (MIBG) scintigraphy, although the preferred modality for neuroblastoma staging, was not available in the local setting; therefore, fluorodeoxyglucose (FDG)-PET was used as an alternative for postoperative assessment, which demonstrated sheet-like thickening and granulation tissue at the surgical site with low-grade FDG uptake, consistent with postoperative changes. Mild uptake was also noted in the right pleura, diaphragm, and chest wall muscles. No metabolically active lesions suggestive of residual or metastatic disease were identified (Figure 4).

**Figure 4:**
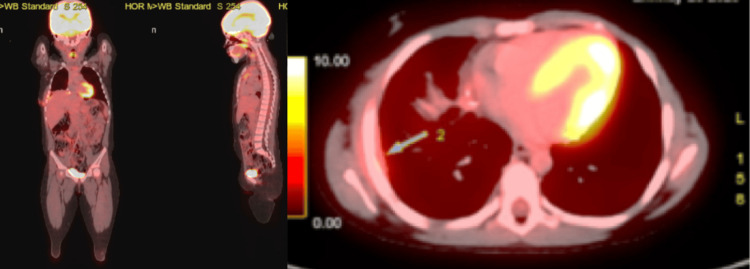
PET/CT findings after right paracardiac mass excision. Postoperative scan showing sheet-like thickening with low-grade fluorodeoxyglucose (FDG) uptake at the surgical site, along with mild FDG uptake in the right costal and basal pleura, diaphragmatic, and chest wall muscles, consistent with early postoperative changes. No other metabolically active lesions are identified.

Based on the absence of metastatic disease, favorable histology, and confinement of the tumor to the thoracic region without image-defined risk factors, the tumor was classified as International Neuroblastoma Risk Group (INRG) stage L1.

At the six-month follow-up, she remained well, with no clinical or radiological evidence of recurrence, normal growth and development, and no need for adjuvant chemotherapy or radiotherapy. She continues to be followed closely by the oncology and pulmonology teams, with regular surveillance imaging. Overall, her prognosis is favorable given the complete surgical resection, localized stage, absence of *MYCN* amplification, and favorable histological subtype of neuroblastoma.

## Discussion

This case illustrates an uncommon presentation of thoracic neuroblastoma in a young child presenting with recurrent wheezing and respiratory symptoms. Importantly, the initial chest radiograph demonstrated a persistent right paracardiac opacity, which was initially interpreted as consolidation but, in retrospect, represented an early radiologic clue to an underlying structural thoracic lesion. Although wheeze is most commonly associated with asthma or viral lower respiratory tract infections, structural causes, including mediastinal masses, should be considered when symptoms are persistent, atypical, or when radiologic abnormalities fail to resolve as expected.

Previous reports of thoracic neuroblastoma have described a range of respiratory manifestations, most commonly respiratory distress and wheezing (Table 2). This case highlights the potential for diagnostic delay when initial radiologic findings are subtle or attributed to more common respiratory conditions.

**Table 2:** Thoracic neuroblastoma cases presenting with wheezing: literature review

Author	Year	Country	Number of Cases	Age	Clinical Picture	Resolution
Bilalaga et al. (Current Case)	2026	United Arab Emirates/Egypt	1 case	3 years	Recurrent wheezy chest and lower respiratory tract infections, initially managed as recurrent viral-induced wheeze/asthma	Successful surgical excision of the mass with complete resolution of cough, wheezing, and chest congestion
Fraga et al. [6]	2010	United Kingdom and Brazil	5/43 cases	3 years (average age)	Wheezing, raised catecholamines, high Lactate Dehydrogenase and normal ferritin	38 cases were treated with posterolateral thoracotomy and 5 with thoracoscopy
Simpson and Campbell [7]	1991	Australia	Up to 5 cases	<2 years old (average age)	Wheezing, stridor and respiratory difficulty with a raise in catecholamines	good prognosis noted
Scarfone [8]	2000	United States	1 case	14 months	Wheezing, difficulty breathing, clear rhinorrhoea, nasal congestion, nonproductive cough, fever, and diminished oral intake	Child underwent thoracotomy with excision of most of the mass

Author	Year	Country	Number of Cases	Age	Clinical Picture	Resolution
Bilalaga et al. (Current Case)	2025	United Arab Emirates/Egypt	1 case	3 years	Recurrent wheezy chest and lower respiratory tract infections, initially managed as recurrent viral-induced wheeze/asthma	Successful surgical excision of the mass with complete resolution of cough, wheezing, and chest congestion
Fraga et al. [6]	2010	United Kingdom and Brazil	5/43 cases	3 years (average age)	Wheezing, raised catecholamines, high Lactate Dehydrogenase and normal ferritin	38 cases were treated with posterolateral thoracotomy and 5 with thoracoscopy
Simpson and Campbell [7]	1991	Australia	Up to 5 cases	<2 years old (average age)	Wheezing, stridor and respiratory difficulty with a raise in catecholamines	good prognosis noted
Scarfone [8]	2000	United States	1 case	14 months	Wheezing, difficulty breathing, clear rhinorrhoea, nasal congestion, nonproductive cough, fever, and diminished oral intake	Child underwent thoracotomy with excision of most of
Author	Year	Country	Number of Cases	Age	Clinical Picture	Resolution
Bilalaga et al. (Current Case)	2025	United Arab Emirates/Egypt	1 case	3 years	Recurrent wheezy chest and lower respiratory tract infections, initially managed as recurrent viral-induced wheeze/asthma	Successful surgical excision of the mass with complete resolution of cough, wheezing, and chest congestion
Fraga et al. [6]	2010	United Kingdom and Brazil	5/43 cases	3 years (average age)	Wheezing, raised catecholamines, high Lactate Dehydrogenase and normal ferritin	38 cases were treated with posterolateral thoracotomy and 5 with thoracoscopy
Simpson and Campbell [7]	1991	Australia	Up to 5 cases	<2 years old (average age)	Wheezing, stridor and respiratory difficulty with a raise in catecholamines	good prognosis noted
Scarfone [8]	2000	United States	1 case	14 months	Wheezing, difficulty breathing, clear rhinorrhoea, nasal congestion, nonproductive cough, fever, and diminished oral intake	Child underwent thoracotomy with excision of most of the mass

Neuroblastic tumors, including neuroblastoma and ganglioneuroblastoma, represent a histopathological spectrum, and a previous case report has described thoracic ganglioneuroblastoma masquerading as viral-induced wheeze in a three-year-old girl [9]. Neuroblastoma comprises approximately 8-10% of pediatric cancers and contributes to about 15% of childhood cancer-related deaths in recent global epidemiologic analyses [10]. Reported frequencies in the literature range from approximately 11-26% of tumors in some cohorts [11].

The mechanism underlying wheezing in thoracic neuroblastoma is most plausibly related to airway compression. In our case, imaging demonstrated mass effect on the right main bronchus, supporting a structural explanation. The resolution of respiratory symptoms following surgical resection further strengthens this association. It is also possible that local cytokines can produce this clinical picture. This, however, remains to be studied.

While wheezing is typically associated with asthma or viral bronchiolitis, structural or neoplastic causes should be considered when the response to therapy is inadequate or when symptoms persist beyond expected patterns. Although the initial response to medical therapy may suggest a superimposed respiratory condition, the recurrence of symptoms, persistence of radiologic abnormalities, and subsequent resolution following tumor resection suggest a possible association between the mediastinal mass and the clinical presentation. The persistence of radiographic findings warranted further evaluation; therefore, a notable aspect of this case is the delay in definitive diagnosis despite early radiologic abnormalities. This delay was primarily related to deferral of recommended investigations, including advanced imaging, during multiple clinical encounters. This case, therefore, underscores the importance of follow-up imaging when pulmonary opacities do not resolve as expected despite appropriate medical therapy.

Molecular risk stratification in neuroblastoma is primarily driven by *MYCN* amplification status, which remains one of the most important adverse prognostic markers [12]. In this case, absence of *MYCN* amplification, together with favorable histology and localized disease, supported a favorable prognostic profile.

Contemporary neuroblastoma risk stratification is based on integrated clinical, pathological, and molecular features, including INRG staging, histological differentiation, and key genetic alterations. Thoracic neuroblastomas are generally associated with a more favorable prognosis compared with other primary sites. This is thought to relate to earlier clinical detection due to respiratory symptoms, a higher proportion of biologically favorable tumors, and a tendency toward maturation. In localized disease (INRG stage L1), complete surgical resection is often curative with excellent outcomes [2]. The present case demonstrated all of these favorable prognostic features, including complete excision and absence of metastatic disease.

The present case emphasizes the need for careful reassessment in pediatric patients with recurrent respiratory symptoms that deviate from typical clinical patterns. Early imaging review, attention to persistent asymmetry, and multidisciplinary collaboration are essential to avoid delay in definitive diagnosis.

## Conclusions

Persistent or atypical respiratory symptoms in children, particularly when associated with non-resolving radiologic abnormalities, should prompt evaluation for underlying intrathoracic pathology, including mediastinal masses. While delayed diagnosis in this case did not adversely affect oncologic outcome, it was associated with prolonged symptoms and delayed definitive management. Early recognition of persistent imaging abnormalities can facilitate earlier diagnosis and reduce morbidity.
